# MASP-3 is the exclusive pro-factor D activator in resting blood: the lectin and the alternative complement pathways are fundamentally linked

**DOI:** 10.1038/srep31877

**Published:** 2016-08-18

**Authors:** József Dobó, Dávid Szakács, Gábor Oroszlán, Elod Kortvely, Bence Kiss, Eszter Boros, Róbert Szász, Péter Závodszky, Péter Gál, Gábor Pál

**Affiliations:** 1 Institute of Enzymology, Research Centre for Natural Sciences, Hungarian Academy of Sciences, Magyar tudósok körútja 2, H-1117, Budapest, Hungary; 2 Department of Biochemistry, Eötvös Loránd University, Pázmány Péter sétány 1/C, H-1117, Budapest, Hungary; 3 Institute for Ophthalmic Research, University of Tübingen, Röntgenweg 11, 72076 Tübingen, Germany; 4 Department of Hematology, Institute of Internal Medicine, University of Debrecen, Nagyerdei krt. 98, H-4032, Debrecen, Hungary

## Abstract

MASP-3 was discovered 15 years ago as the third mannan-binding lectin (MBL)-associated serine protease of the complement lectin pathway. Lacking any verified substrate its role remained ambiguous. MASP-3 was shown to compete with a key lectin pathway enzyme MASP-2 for MBL binding, and was therefore considered to be a negative complement regulator. Later, knock-out mice experiments suggested that MASP-1 and/or MASP-3 play important roles in complement pro-factor D (pro-FD) maturation. However, studies on a MASP-1/MASP-3-deficient human patient produced contradicting results. In normal resting blood unperturbed by ongoing coagulation or complement activation, factor D is present predominantly in its active form, suggesting that resting blood contains at least one pro-FD activating proteinase that is not a direct initiator of coagulation or complement activation. We have recently showed that all three MASPs can activate pro-FD *in vitro*. In resting blood, however, using our previously evolved MASP-1 and MASP-2 inhibitors we proved that neither MASP-1 nor MASP-2 activates pro-FD. Other plasma proteinases, particularly MASP-3, remained candidates for that function. For this study we evolved a specific MASP-3 inhibitor and unambiguously proved that activated MASP-3 is the exclusive pro-FD activator in resting blood, which demonstrates a fundamental link between the lectin and alternative pathways.

The complement system^1^,^2^ is an integral part of the innate immune response, and it is responsible for the elimination of invading microorganisms and altered self-structures. It contributes to the immune homeostasis and through its classical pathway (CP), which is primarily triggered by antigen-antibody complexes, it is also linked to the adaptive immune system. Complexes of the lectin pathway (LP) are structurally similar to C1^3^, the first component of the CP, and both pathways lead to formation of the same C3 convertase, C4b2a. The alternative pathway (AP), which can be activated on its own, serves as an amplification loop for all three pathways^4^. C3 convertases cleave C3, generating C3b, which then binds factor B (FB). The pro-convertase, C3bB, is cleaved by factor D (FD)^5^ yielding the AP C3 convertase, C3bBb, which in turn generates even more C3b molecules. C3b carries a newly exposed thioester bond and can covalently attach to the activating surface, where it serves as an opsonin. Further activation of the complement cascade generates a membrane attack complex resulting in the lysis of certain pathogens.

The LP is composed of a heterogeneous mixture of complexes, each containing one pattern recognition molecule (PRM) and one or two dimers of associated serine proteases and other related non-enzymatic proteins^6^,^7^. At least five different LP PRMs have been described: mannose-binding lectin (MBL), three ficolins, and collectin-LK^3^,^8^. The LP is triggered when the PRMs bind to surfaces displaying carbohydrate arrays or certain acetylated compounds. Two MBL-associated serine proteases, MASP-1 and MASP-2, elicit the enzymatic signal upon LP activation. Both are essential: MASP-1 autoactivates first, then it activates MASP-2^9^,^10^,^11^,^12^, and while both enzymes cleave C2, only MASP-2 cleaves C4. The roles of the third associated protease, MASP-3, and the other associated proteins, MAp19 (aka sMAP, MAP-2) and MAp44 (aka MAP-1), have been unclear, but initially all three were considered as negative LP regulators^13^,^14^,^15^. Mutations of the *MASP1* gene that specifically affect MASP-3 function^16^,^17^ cause developmental abnormalities (called the 3MC syndrome) suggesting that MASP-3 has an important physiological role, which might even be unrelated to the complement system.

For a long time, complement FD was presumed to be intracellularly activated at the site of its synthesis^18^. This assumption was based on observations that only active FD was purified from normal blood^19^,^20^, and predominantly active FD was detected in mammalian cell cultures^21^,^22^. However, in *MASP1* knock-out mice lacking both MASP-1 and MASP-3 no AP activity was observed, and proenzyme FD (pro-FD) was detected in the serum^23^. Both MASP-1^23^ and later MASP-3 were implicated as pro-FD activators, and even proenzyme MASP-3 was considered to play this role^24^. In marked contrast, AP activity was detected in the serum of a MASP-1/3 deficient 3MC patient^12^, questioning the involvement of any of these enzymes in pro-FD activation in humans. The complexity of the problem increased when the sera of MASP-1 and −3 deficient 3MC patients were shown to contain only (or predominantly) pro-FD^25^. The mechanism of AP activity in these patients has remained unresolved^26^.

To clarify the above controversy we initiated a comprehensive study, and in a recent paper presented our first findings as follows: i) normal resting human blood has a pro-FD activating capacity; ii) none of the MASP proenzymes can activate pro-FD, but iii) all three activated MASPs are able to activate pro-FD *in vitro*; and iv) pro-FD activating capacity of resting human blood is resistant to selective MASP-1 and MASP-2 inhibition. Based on these we concluded that out of the three MASPs, only MASP-3 is a plausible pro-FD activator in normal resting human blood^27^. Nevertheless, lacking a specific MASP-3 inhibitor we could not directly prove this potential MASP-3 function or exclude the possibility that many different pro-FD activating proteases act simultaneously in resting human blood.

To quantitatively determine the contribution of MASP-3 to pro-FD activation in resting blood, we developed a specific, high-affinity MASP-3 inhibitor via directed protein evolution using phage display. With our monospecific inhibitor we unequivocally identified an essential physiological role of MASP-3 in normal human resting blood.

## Results

### A novel MASP-3 inhibitor developed through directed protein evolution

**Figure 1:**
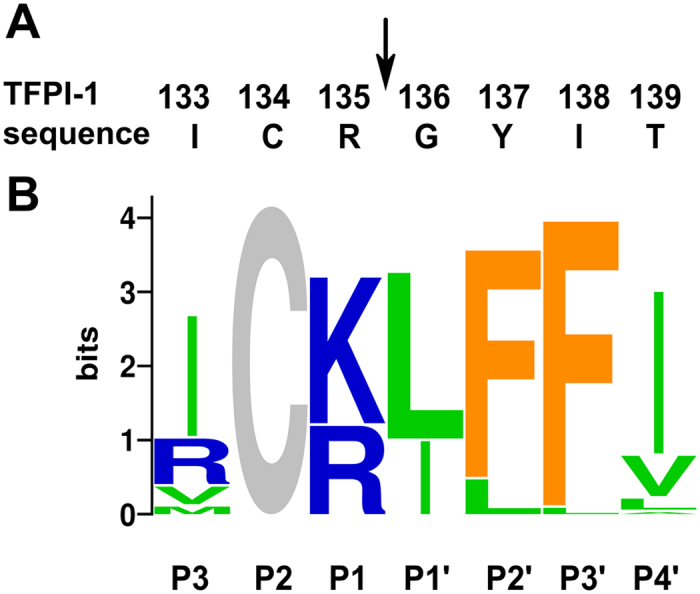
Development of the MASP-3 specific inhibitor, TFMI-3. (**A**) The TFPI-1 domain 2 (TFPI-D2) sequence around the P1 (135) position. The numbering corresponds to the full length protein sequence from the UniProt database. (**B**) Codon-bias normalized sequence logo of MASP-3-selected unique clones from the TFPI-D2 phage library. Position heights represent the degree of conservation. The nonrandomized Cys residue shows the maximum height. Letter heights indicate normalized amino acid frequencies. Colors reflect chemical properties.

We developed a novel, high-specificity MASP-3 inhibitor starting from a natural proteinase inhibitor domain. The second domain (residues 121–178) of human Tissue Factor Pathway Inhibitor-1 (TFPI-1; UniProt ID P10646), hereafter referred to as TFPI-D2, was displayed on M13 phage as an N-terminal fusion to the major coat protein, p8. Phage carrying TFPI-D2 did not bind to immobilized MASP-3. A library was generated by fully randomizing six positions P3, P1, P1′, P2′, P3′ and P4′^28^ of the protease binding loop while keeping the structurally indispensable Cys at P2 (Fig. 1A). The library of 5 × 10^8^ clones was selected on the immobilized catalytic fragment of active MASP-3 containing the CCP1-CCP2-SP domains (hereafter referred to as MASP-3cf). Based on phage-ELISA all clones from the third selection cycle could bind MASP-3cf. DNA of 71 clones was sequenced to reveal the sequence pattern that allows for MASP-3 binding.

### A characteristic binding loop sequence pattern emerged

DNA sequencing identified 31 unique clones (Table S1). The characteristic pattern emerging from the individual sequences is illustrated as codon-bias normalized protein sequence logo^29^ shown on Fig. 1B. The pattern is more hydrophobic than any other binding loops we have evolved against various proteinases^9^,^30^,^31^.

### The evolved consensus defines a highly specific MASP-3 inhibitor

In phage display studies, normalized amino acid frequencies usually correlate with binding energy contributions of individual amino acid residues^32^,^33^,^34^,^35^,^36^,^37^. On the basis of this notion we produced the presumably tightest binding TFPI-D2 variant carrying the selected consensus sequence ICKLFFI between the P3-P4′ positions of the canonical protease binding loop (note that P2 Cys was not randomized). The variant was expressed in *E. coli*, purified to homogeneity, and its inhibitory potency was assessed on MASP-1cf, MASP-2cf and MASP-3cf (Table 1).

The variant turned out to be a potent MASP-3 inhibitor with an 11.1 ± 1.4 nM equilibrium inhibitory constant (K_I_), while it is almost inactive on MASP-2cf (K_I_ is 75 000 ± 3 000 nM) and totally inactive on MASP-1cf. Because of this specificity profile we named the variant ‘TFPI-based MASP-3 Inhibitor’, hereafter in brief as TFMI-3. We produced and tested the parent molecule, wild-type TFPI-D2 as a control and found that it does not inhibit MASP-3 and MASP-1 at all, and inhibits MASP-2 rather weakly with a K_I_ of 318 ± 11 nM (Table 1) consistently with previous observations^38^.

The MASP-3cf/TFMI-3 interaction was also tested in the absence of calcium ions and the K_I_ was found to be 9.9 ± 0.6 nM suggesting that Ca^2+^ ions do not affect the interaction.

### TFMI-3 does not bind zymogen MASP-3

**Figure 2:**
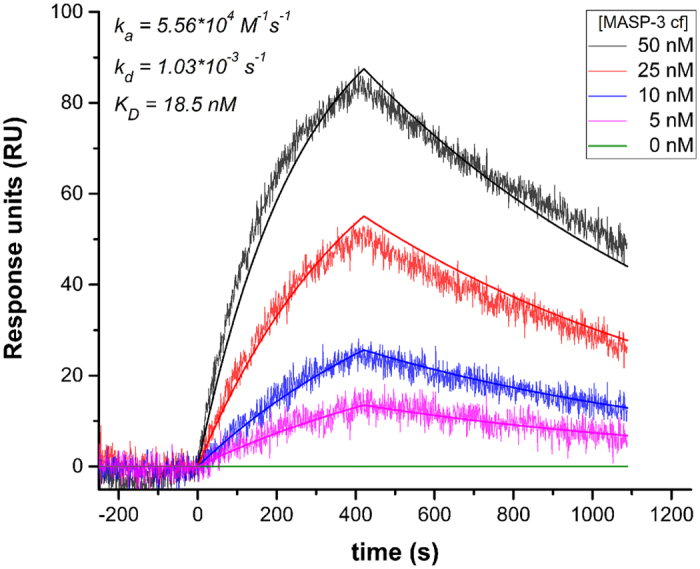
Interaction of TFMI-3 and MASP-3 determined by Surface Plasmon Resonance. MASP-3cf was injected over TFMI-3_HA immobilized on anti-HA antibody-coated GLM sensor chip as described in “Experimental Procedures”. Result of one of the three independent runs is shown as sensograms corresponding to five parallel analyte injections of different MASP-3cf concentrations. Smooth lines represent global fit of the experimental data to a 1:1 Langmuir model.

As proenzyme MASP-3 has no measurable activity, enzyme kinetic studies cannot test the binding of TFMI-3 to zymogen MASP-3. We performed surface plasmon resonance (SPR) experiments to test it and also to provide an independent K_D_ value for the interaction between TFMI-3 and active MASP-3. Moreover, while the equilibrium enzyme inhibition studies did not deliver kinetic rate constants for complex formation and dissociation, SPR provides these values as extra information. The inhibitor carrying a C-terminal HA (hemagglutinin peptide) tag (TFMI-3_HA) was immobilized in oriented manner on an anti-HA antibody coated sensor chip. Serial dilutions of active or zymogen MASP-3 were injected in separate experiments. Data generated upon active MASP-3 injections were readily fitted to the simplest 1:1 Langmuir binding model as illustrated by the sensograms of a typical run (Fig. 2). Three independent runs yielded a 17.7 ± 2.0 nM dissociation binding constant (K_D_), which compares well to the 11.1 ± 1.4 nM K_I_ determined by the solution phase enzyme inhibition assays. The association and dissociation rate constant values were 5.25 ± 0.48 × 10^4 ^M^−1^s^−1^ and 9.30 ± 1.40 × 10^−4 ^s^−1^, respectively.

Importantly, TFMI-3 did not bind zymogen MASP-3 at all even at 10 μM concentration of the proenzyme.

### TFMI-3 does not interfere with blood coagulation

Blood coagulation is an intricate and delicately regulated cascade system involving several trypsin-like proteases. To assess the selectivity of our unique inhibitor, we tested whether TFMI-3 affects blood clotting in three standard coagulation assays, the thrombin time (TT), prothrombin time (PT) and the activated partial thromboplastin time (APTT). TFMI-3 was applied in a fivefold serial dilution with the highest final concentration being 36 μM, which is ~3000-fold higher than the K_I_ value of TFMI-3 on MASP-3cf. Yet, even at this highest concentration TFMI-3 had only negligible if any effect. The corresponding blood clotting times in seconds are as follows, TT: control 16.4, TFMI-3 15.8; PT: control 10.7, TFMI-3 11.0; APTT: control 37.3, TFMI-3 38.6. The three assays altogether imply that TFMI-3 does not inhibit any of the six blood coagulation proteases: thrombin, fVIIa, fIXa, fXa, fXIa and fXIIa. It also shows that at least in these standard assays MASP-3 has no contribution to blood coagulation.

### TFMI-3 does not inhibit any of the three complement pathways in standard assays

**Figure 3:**
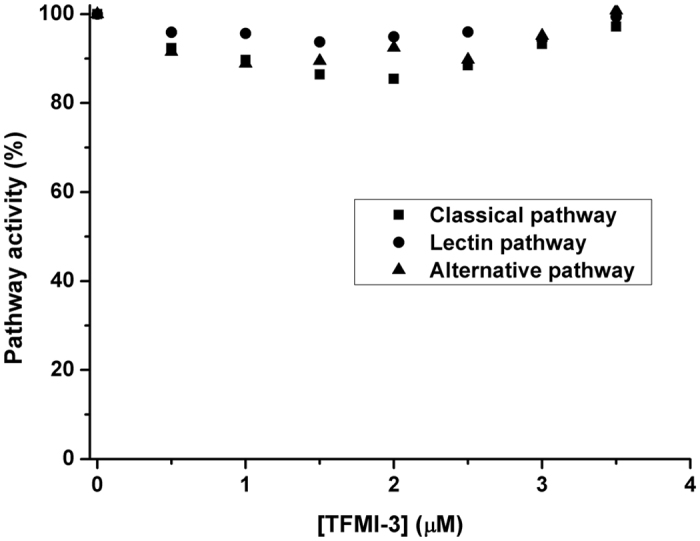
Lack of inhibitory effect of TFMI-3 on the three complement pathways. TFMI-3 was applied in 0–3.5 μM in standard complement activation assays (Wieslab). No significant inhibition of any of the three pathways, CP (■), LP (●) or AP (▲) was observed in normal human serum. Typical curves from 3 parallel assays are shown where each data point represents the mean of 3 repeats. SD bars are not shown to allow distinction of the symbols.

The Wieslab COMPL 300 (WIELISA) assay individually triggers the classical, the lectin and the alternative complement pathway and measures the amount of the final product cleaved C9^39^. We applied this assay to test the inhibitory properties of TFMI-3 in part to further characterize its selectivity. TFMI-3 proved to be extremely selective as it did not interfere with any of the three complement pathways even at a 3.5 μM final concentration, which is over 200-fold higher than its K_I_ value on MASP-3 (Fig. 3). The lack of any inhibitory effect of TFMI-3 in these assays indicated two important things: i) MASP-3 does not contribute to the activation of the following enzymes: FB, MASP-1, MASP-2, C1r, C1s or any of the convertases; and ii) TFMI-3 does not inhibit any of the above mentioned proteases or their activators. Namely, neither the zymogen nor the activated forms of FD, FB, MASP-1 and MASP-2, C1r, C1s, or the C3 and C5 convertases are inhibited at a 3.5 μM TFMI-3 concentration, where MASP-3 is completely inhibited. This opened the question whether the proteolytic activity of MASP-3 has any biological function in human blood.

### MASP-3 is the only plasma proteinase binding to TFMI-3

To identify all plasma proteinases that are able to bind TFMI-3 we performed affinity pull-down assays. TFMI-3_HA was incubated with plasma and captured by anti-HA antibody coated magnetic beads. Competitive elutions were performed in two consecutive steps by incubating the beads with large excess of HA peptides for 5 and then for 90 minutes. Proteins bound to the inhibitor were identified by mass-spectrometry. Beads carrying a nonfunctional inhibitor variant, TFMI-3_K135E_HA and beads carrying no inhibitor at all served as controls. Only two proteins were exclusively associated with the functional TFMI-3-HA bait: the targeted protease MASP-3, and Ficolin-3 (H-ficolin), the most abundant PRM associated with MASP-3^40^. After the longer, 90 minutes elution, these two proteins were identified with five and seven unique tryptic peptides, respectively (Table 2 and Table S2). MASP-1 and MASP-3 share their first 5 domains but have a distinct C-terminal SP domain. Importantly, four out of the five MASP peptides reside within the unique MASP-3 SP domain (Fig. S1). The assay verified that MASP-3 is the only plasma proteinase inhibited by TFMI-3.

### MASP-3 is the exclusive activator of pro-FD in normal human plasma

Using purified recombinant proteins we have recently shown that all three activated MASPs are able to process pro-FD *in vitro*^27^. In that study we performed plasma assays as well, and using our previously developed MASP-1 and MASP-2 inhibitors we proved that neither MASP-1 nor MASP-2 acts as pro-FD activator in normal resting human blood. The apparent discrepancy of the two tests can be resolved by the notion that resting blood (and therefore the plasma) does not contain active MASP1 and MASP-2. These enzymes are present either in zymogen or in activated but serpin-inhibited form. In contrast, MASP-3 has no natural inhibitor identified to date, therefore the possibility that in resting blood MASP-3 functions as a pro-FD activator remained open^27^.

**Figure 4:**
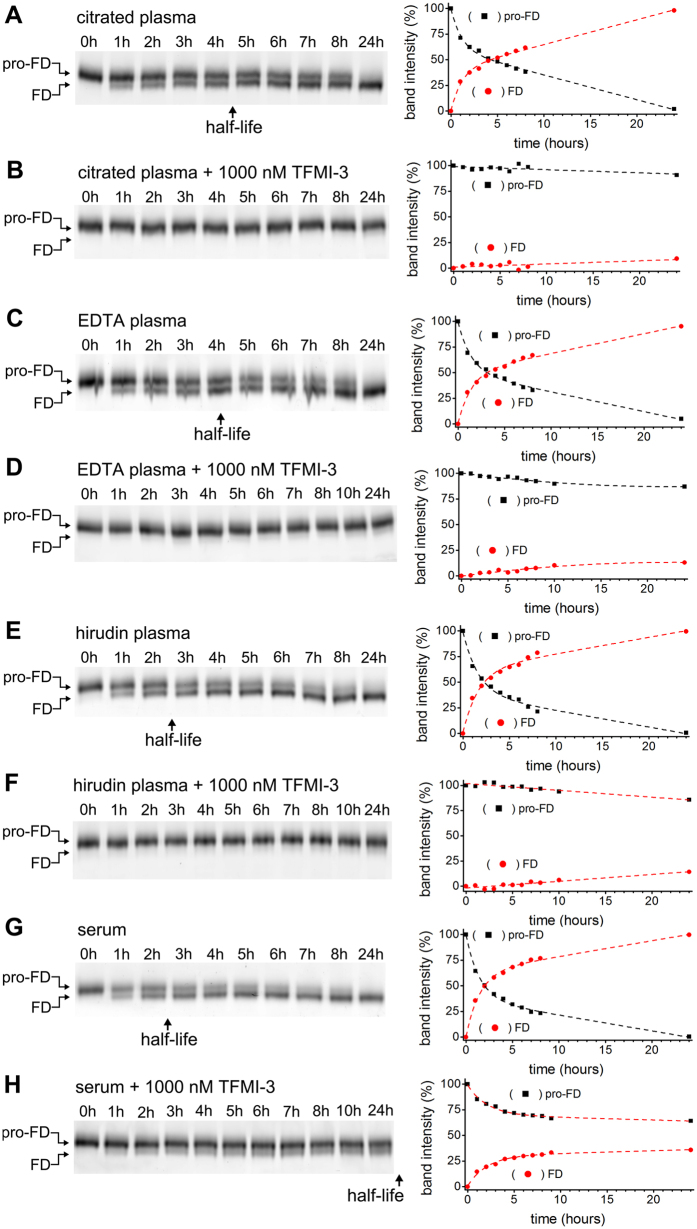
Inhibition of pro-FD activation by TFMI-3. Conversion of Cy3-labeled pro-FD to Cy3-FD (referred to as activation) in different types of normal human blood preparations was detected by reducing SDS-PAGE followed by fluorimetric scanning and densitometry. Representative gels out of at least 2 parallels are shown on the left side. On the right side of each panel densitometric analysis of the pro-FD and the FD bands of the same gel is shown. The intersection point of the dashed trend lines gives the half-life of pro-FD. Calculated half-lives are found in s 3. (A) Activation of Cy3-pro-FD in citrated normal human plasma. (**B**) Lack of significant activation of Cy3-pro-FD in citrated normal human plasma in the presence of 1000 nM TFMI-3. (**C**) Activation of Cy3-pro-FD in normal human EDTA plasma. (**D**) Lack of significant activation of Cy3-pro-FD in normal human EDTA plasma in the presence of 1000 nM TFMI-3. (**E**) Activation of Cy3-pro-FD in hirudin-treated normal human plasma. (**F**) Lack of significant activation of Cy3-pro-FD in normal human hirudin plasma in the presence of 1000 nM TFMI-3. (**G**) Activation of Cy3-pro-FD in normal human serum. (**H**) Delayed activation of Cy3-labelled pro-FD in normal human serum in the presence of 1000 nM TFMI-3.

We have repeated the previously developed pro-FD activation assays, but this time using our novel MASP-3 specific TFMI-3. The MASP-3 inhibitor completely blocked pro-FD activation in three types of normal human plasma preparations we used as resting blood models (Fig. 4 and Table 3). Based on the measured 11 nM K_I_ value, at an estimated 10–20 nM active MASP-3 plasma concentration^27^, 100 nM TFMI-3 would result in approximately 10%, while 1 μM TFMI-3 would result in about 1% residual MASP-3 activity. The results (Table 3) are in good agreement with these calculations.

At 1 μM concentration TFMI-3 nearly completely blocked pro-FD activation in all types of plasma preparations irrespective of the anticoagulant applied. This showed that in these resting blood models MASP-3 is the exclusive pro-FD activator.

However, we found that pro-FD activation was significantly faster in the Ca^2+^-containing hirudin-treated plasma than in the Ca^2+^-depleted citrated or EDTA-treated plasma (Table 3). This indicates that either directly or indirectly Ca^2+^ facilitates MASP-3 driven pro-FD activation. To test the potential direct Ca^2+^ effect, we used purified recombinant MASP-3cf and pro-FD and in parallel experiments measured the rate of pro-FD activation in the presence or absence of Ca^2+^ (Fig. S2). There was no difference at all demonstrating that Ca^2+^ ions do not affect the interaction of the two proteinases.

Besides plasma assays as resting blood models, we also performed serum experiments as coagulated blood models. The serum experiments showed that coagulation yields an enzyme pool that is absent from plasma. This extra pool was detected through the slow conversion of pro-FD to FD due to an enzyme activity uninhibited even at 1 μM of TFMI-3.

## Discussion

MASP-3 was identified 15 years ago as the third MBL associated protease^13^ and as an alternative splice product of the *MASP1* gene. MASP-3 differs from MASP-1 only in the serine protease (SP) domain. In spite of having an SP domain, recombinant MASP-3 was found to down-regulate C4 deposition efficiency of MBL-MASP complexes; hence MASP-3 was assumed to have an inhibitory role^13^. A novel MASP-3 function has recently been discovered through genetic studies. Several mutations in the *MASP1* gene that apparently diminish MASP-3 enzyme activity were reported to cause the 3MC (Malpuech-Michels-Mingarelli-Carnevale) syndrome, a severe developmental disorder^16^,^17^,^41^,^42^.

The sera of 3MC patients lacking both MASP-1 and MASP-3 were shown to provide AP activity, albeit at a lower than normal level^12^,^17^. In contrast, sera of KO mice lacking both MASP-1 and MASP-3 provided no detectable AP activity, and contained pro-FD instead of FD^23^. The puzzle became even more complex when it was shown that as in the case of KO mice, the sera of MASP-1/3 deficient 3MC patients also contained predominantly pro-FD^25^.

To untangle this problem and to dissect the specific roles of the three MASPs in pro-FD maturation we developed a direct assay measuring proteolytic conversion of exogenously provided recombinant, fluorescently labeled pro-FD in human blood samples^27^.

Using specific *in vitro* evolved MASP-1 and MASP-2 inhibitors we have successfully revealed the longtime hidden essential function of MASP-1 in LP initiation^9^,^10^. We made good use of the same approach when we showed that in resting blood neither MASP-1, nor MASP-2 acts as pro-FD activator^27^. When we applied SGMI-2, which is a nanomolar MASP-2 inhibitor but also a poor MASP-3 inhibitor, in micromolar concentration, it provided partial MASP-3 inhibition and lowered the pro-FD conversion rate in resting blood. Our results suggested that activated MASP-3 could be a pro-FD activator^27^. However, without a specific MASP-3 inhibitor we could not unambiguously prove this, and were also unable to determine the exact extent of MASP-3′s contribution to pro-FD activation.

To identify the exact roles of MASP-3 we developed a novel MASP-3 inhibitor via directed evolution. There are many structurally distinct families of canonical serine protease inhibitors and the inhibitor scaffold can greatly affect the evolvable specificity of the inhibitor^9^,^30^,^43^. We tried various scaffolds but only the one reported here provided a specific inhibitor. The evolved binding loop pattern is uniquely hydrophobic, particularly at the primed side (P1′-P4′ LFFI). It is in good line with the apolar nature of the MASP-3 substrate binding groove revealed by the recent crystal structure of a complex formed by MASP-3 and the pan-specific bacterial protease inhibitor, ecotin^44^. The structure of MASP-3 in that complex (PDB ID 4IW4) reveals several hydrophobic residues including W481 and F482 (precursor numbering), which might interact with the P2′P3′ FF motif in TFMI-3. The primed side of the pro-FD activation site (P1′-P4′ ILGG) contains small hydrophobic residues. This sequence is certainly compatible with the MASP-3 substrate binding cleft, which is able to accommodate the much bulkier hydrophobic P1′-P4′ LFFI segment of TFMI-3.

TFMI-3 has high affinity (K_I_ ~11 nM) for MASP-3, while it does not bind to zymogen MASP-3 at all. TFMI-3 has outstanding specificity, as the only protease it extracts from human plasma is MASP-3. In perfect line with this, TFMI-3 does not inhibit any of the active or zymogen proteinases involved in the standard coagulation and complement activation assays.

These assays also demonstrated that MASP-3 has no detectable role in blood coagulation, and except for its fundamental role identified in this study, it does not promote complement activation either. Applying TFMI-3 to three kinds of normal human plasma preparations at 1 μM, which provides about 99% MASP-3 inhibition, we found that activation of exogenously added pro-FD was indeed practically blocked.

On the other hand, when TFMI-3 was applied in a standard AP activity assay, no reduction of C3 deposition was observed. Since endogenous FD had been already activated in normal resting blood prior to plasma or serum preparation, lack of any effect of MASP-3 inhibition in traditional AP tests is self-explanatory.

Pro-FD activation was clearly faster in hirudin plasma than in citrated or EDTA plasma, implying that Ca^2+^ plays a positive role in pro-FD activation. The rate of Cy3-pro-FD conversion in hirudin treated plasma was almost as high as that in serum, but importantly, unlike in serum, in hirudin-treated plasma TFMI-3 completely inhibited the process. Based on the thoroughly demonstrated mono-specificity of TFMI-3 the increased pro-FD activity must be due to increased MASP-3 activity. This in turn could be due to two mutually nonexclusive causes. One is a direct effect of the calcium ions on the MASP-3 driven pro-FD activation either by enhancing the catalytic efficiency of MASP-3, or by making pro-FD a more suitable MASP-3 substrate. However, using purified recombinant enzymes we demonstrated that Ca^2+^ does not directly affect the rate of MASP-3 driven pro-FD activation. The second possible cause is an indirect effect of Ca^2+^ most probably leading to mobilization of MASP-3-activating proteinases. Our results are in line with this second proposed mechanism.

Calcium depletion is a powerful general approach to block the activation of almost all plasma proteinases. In comparison, hirudin is a highly specific direct thrombin inhibitor, which does not inhibit any calcium-dependent, but thrombin-independent activation of proteinases. We suggest that compared to the decalcified plasma preparations the hirudin-treated plasma contains extra proteinase species. These extra enzymes do not directly activate pro-FD as our mono-specific MASP-3 inhibitor completely blocked pro-FD cleavage in the hirudin-treated plasma. On the other hand these extra proteases may generate additional activated MASP-3. This, in turn implies that in resting blood only a portion of the MASP-3 pool is activated, while the rest may still be in zymogen form that can be mobilized.

We used serum as a model of coagulated blood. Our experiments confirmed that at least one pro-FD activating enzyme other than MASP-3 is present in coagulated blood, as low level residual pro-FD conversion was observed in the serum at a TFMI-3 concentration providing complete MASP-3 inhibition. On the other hand, in hirudin-treated plasma, where thrombin is specifically inactivated, no residual pro-FD activation was observed when MASP-3 was completely blocked. These observations together identify thrombin as the most likely backup pro-FD activator, but allow that proteinases activated through thrombin might also play such a role. In fact, thrombin was shown to be able to activate pro-FD *in vitro*^27^,^45^. Moreover, MASP-1 and MASP-2 were also shown to be activated by platelets and fibrin during coagulation^46^ potentially generating even more backup enzymes.

Coagulation enzymes obviously cannot represent the physiologic pro-FD activation route in normal resting blood. On the other hand, coagulation might temporarily boost the pro-FD activation rate in normal blood. Moreover, a baseline level activation of coagulation enzymes might also provide a low-efficiency backup route in MASP-3 deficient blood. We suggest that the low level active FD and the detectable albeit subnormal level of AP activation in the sera of 3MC patients is due to the presence of active coagulation enzymes generated upon serum preparation.

Western blots of plasma MBL-MASP complexes separated on reducing SDS PAGE always detected the light chain of activated MASP-3, but not the single-chain zymogen form^13^,^47^. As MASP-3 apparently lacks autoactivation capacity^48^, the observed abundance of the activated form is unexpected. Western blots, however, do not show whether MASP-3 became activated prior to, or during isolation. Our results imply that a significant proportion of MASP-3 must be present in the active form even in resting blood, as only active MASP-3 can activate pro-FD^27^, and our MASP-3 inhibitor binds only the active enzyme. The ratio of the zymogen and active forms of MASP-3 in normal human resting blood is yet to be determined and the physiologic MASP-3 activating mechanism in the resting state also needs to be identified.

Our results unequivocally demonstrate that active MASP-3 is present in normal human resting blood. Unlike most plasma proteinases, MASP-3 appears to lack any physiologic inhibitors. In fact, this should be instrumental for a functional steady state level of MASP-3 activity in resting blood.

Most importantly, we show for the very first time that MASP-3 is the exclusive activator of pro-FD in resting blood. Through this exclusive and central function MASP-3 provides a hitherto unproven fundamental link between the lectin and the alternative complement pathways. This link continuously supplies active FD establishing an immediately available AP activation capacity for the resting blood. MASP-3 is one of the very few serine proteinases that have no natural inhibitor identified to date. This lack of natural inhibitors should be a key factor in explaining the exclusive pro-FD activating role of MASP-3.

We demonstrated that in resting blood, the alternative pathway strictly relies on the presence of an extraneous proteinase, which is a component of the lectin pathway. Our discovery questions whether the alternative pathway can be rightfully considered as an autonomous complement activation route.

## Methods

### Recombinant human MASPs

Active MASP-1cf, active MASP-2cf, zymogen MASP-3cf and active MASP-3cf were produced as described^11^,^27^,^49^,^50^. The constructs encompass the last three domains including the serine protease (SP) domain for each enzyme.

### Construction of the TFPI-D2 library

The vector for displaying TFPI-D2 on the surface of M13 bacteriophage was based on the pSFMI-pro-lib vector^30^, which was modified through general recombinant DNA methods. The original inhibitor-encoding region was replaced with the synthetic gene of TFPI-D2 (from Life Technologies) (Table S3). The resulting pTFPI-D2-pro-lib vector encodes a fusion protein consisting of an N-terminal FLAG tag, TFPI-D2 flanked by Ser/Gly linkers and the p8 coat protein of the M13 bacteriophage (Fig. S3). The FLAG tag enables assessment of display efficiency. The library was produced by two successive Kunkel mutagenesis^51^ steps according to Sidhu *et al*.^52^ using mutagenesis primers listed in Table S3. The phagemid library was electroporated into *E. coli* SS320 cells^52^ to produce the TFPI-D2 phage library.

### Selection and identification of MASP-3 binding clones and generation of the sequence logo

Active MASP-3cf (20 μg/ml) was immobilized on MaxiSorp (Nunc) plates in 10 mM Tris, 10 mM Na-phosphate, 155 mM NaCl, pH 8.0 buffer. Three selection and amplification cycles were performed. Individual clones from the third selection cycle were tested for binding to MASP-3cf in ELISA assays^52^. Clones with signals at least 3-times higher on MASP-3 than on BSA control were selected for DNA sequencing. Protein sequence of clones having unique DNA sequence was analyzed. Amino acid frequencies were normalized to the expected codon frequencies in the NNK (N = A, C, G, T; K = G, T) set of degenerate codons to eliminate codon bias. The normalized amino acid frequencies were used to generate a sequence logo by WebLogo^29^.

### Construction, expression and purification of TFMI-3 variants

TFMI-3 was produced by Kunkel mutagenesis^51^ using the pTFPI-D2-pro-lib vector as template. A C-terminally HA-tagged version of TFMI-3 (TFMI-3_HA) was also created via PCR mutagenesis. A P1 Glu mutant of the HA-tagged inhibitor (TFMI-3_K135E_HA) was created using overlap extension PCR. All primer sequences are listed in Table S3. A synthetic gene (from IDT) encoding the N-terminally His_6_-tagged, C3S, C81S, C86S variant of S100A4 followed by a TEV protease cleavage site and a multi cloning site was cloned into the expression vector pBH4^53^ using NcoI and XhoI. TFMI-3 variant genes were cloned into this vector using BamHI and XhoI (Fig. S4). The construct enabled high level expression of the fusion proteins in *E. coli* Shuffle T7 (NEB, C3026H) cells, purification through Ni-NTA chromatography, and liberation of TFMI-3 through TEV protease processing as follows.

*E. coli* Shuffle T7 (NEB, C3026H) cells transformed with a TFMI-3 variant construct were grown in LB medium at 30 °C. The expression was induced at OD_600_ = 0.8 by 0.4 mM IPTG and was continued O/N at 18 °C. Cells were harvested by centrifugation (5 min, 7500g), resuspended in 1/10 culture volume 50 mM Tris-HCl, 300 mM NaCl, 10 mM imidazole, pH 8.0 buffer (chromatography buffer), and disrupted by sonication. The samples were centrifuged (20 min, 48000 g) and the supernatant loaded onto Ni-NTA column (BioRad). The inhibitors were eluted with chromatography buffer supplemented with 250 mM imidazole. The eluates were dialyzed against chromatography buffer and the fusion protein was processed by His-tagged TEV protease O/N at 30 °C. The samples were reloaded to the Ni-NTA column to remove all His-tagged proteins.

The flow-through was further purified by RP-HPLC on a 250 × 10 mm Phenomenex Jupiter 10u C4 300A column. The inhibitors were lyophilized, dissolved in water and the molar concentrations were determined by spectrophotometry using the extinction coefficients ε_280_ = 4845 M^−1^cm^−1^ for TFMI-3 and ε_280_ = 9315 M^−1^cm^−1^ for the HA-tagged variants. The samples were found to be of correct size by mass spectrometry on an HP1100 HPLC-ESI-MS system (Agilent Technologies).

### Inhibitory constant (K_I_) measurements

The photometric assays and the analyses were done based on Kocsis *et al*.^30^. K_I_ values of TFMI-3 and TFPI-D2 on MASP-1cf, MASP-2cf and MASP-3cf were determined in a 100 μL final assay volume with 20 mM HEPES, 145 mM NaCl, 5 mM CaCl_2_, 0.05% Triton X-100, pH 7.5 buffer on 96-well microtiter plates (Nunc) using a BioTek Synergy H4 microplate reader. The enzymes were co-incubated with serial dilutions of the inhibitors for 2 h at room temperature, then 250 μM final concentration of Z-Lys-SBzl substrate and 500 μM final concentration of 5,5′-Dithiobis(2-nitrobenzoic acid) co-substrate were added. Residual enzyme activity was determined at 410 nm in three parallel measurements. K_I_ of TFMI-3 on MASP-3cf was also determined in a Ca^2+^-free buffer: 20mM HEPES, 150mM NaCl, 0.1mM EDTA, 0.05% Triton-X100, pH 7.4.

### Complement and coagulation assays

Wieslab COMPL 300 (aka WIELISA) assays^39^ from Euro Diagnostica were performed to separately test the effect of TFMI-3 on the three complement activation pathways according to the manufacturer’s protocol with the modifications of Kocsis *et al*.^30^. Three parallels were measured for each data point. Serum activity in the presence of TFMI-3 was expressed as percentage of the corresponding activity in the absence of the inhibitor.

Inhibitory capacity of TFMI-3 to slow down the coagulation process was tested in three standard assays, the thrombin time, testing any direct effects on thrombin; prothrombin time, testing any effects on the extrinsic pathway; and the activated partial thromboplastin time, testing any effects on the intrinsic pathway. Blood was collected from a healthy individual by vein puncture after informed consent. The blood was treated with sodium-citrate (3.8% w/v) and centrifuged. All three assays were performed on the automated instrument Sysmex CA-1500 (Sysmex) with Innovin reagent (Dale Behring, Marburg, Germany).

### TFMI-3_HA fusion protein pull-down assay, mass spectrometry and data analysis

20 μl plasma and 1.75 μg TFMI-3_HA were incubated in 500 μl PBS supplemented with 0.1% Tween-20 (PBST) for 1 h at room temperature. The same amount of plasma without the inhibitor served as absolute control. A modified inhibitor harboring a point mutation (TFMI-3_K135E_HA) eliminating MASP-3 binding function was also incubated with plasma and used to detect putative protein-protein interactions unrelated to MASP-3 (specific control). Next, 50 μl anti-HA magnetic beads (Pierce) were added and the incubation was continued for 20 min at room temperature. The beads were collected, intensively washed, and bound proteins were eluted with two subsequent incubations with 100 μl 1 mg/ml HA-peptide (Pierce) for 5 min and 90 min, respectively.

Precipitated eluates were subjected to in-solution trypsin digestion folsis performed on an UltiMate 3000 RSLCnano HPLC system (Dionex) coupled to an Orbitrap Fusion mass spectrometer (Thermo Fisher Scientific) by a nano-spray ion source. All acquired spectra were processed and analyzed using Mascot (Matrix Science, version 2.5.1) and the human-specific Swiss-Prot database (rel. 2015/11/11) manually complemented with entries corresponding to all alternative splice products of the *MASP1* and *2* genes (including MASP-3) missing from the original database. Mascot was searched with a fragment ion mass tolerance of 0.50 Da and a parent ion tolerance of 10.0 PPM. Carboxymethylation of cysteine was specified in Mascot as a fixed modification. Deamidation of asparagine and glutamine and oxidation of methionine were specified as variable modifications. Scaffold (v4.4.8, Proteome Software Inc.) was used to validate MS/MS based peptide and protein identifications.The mass spectrometry proteomics data have been deposited to the ProteomeXchange Consortium via the PRIDE^54^ partner repository with the dataset identifier PXD003666.

### Surface Plasmon Resonance (SPR) measurements

The SPR measurements were carried out on a ProteOn XPR36 protein interaction array system (Bio-Rad) using a GLM sensor chip. HA Epitope Tag Antibody (clone 2–2.2.14) was purchased from Thermo Fisher Scientific and was covalently immobilized through amine coupling as follows. 80 nM EDC (1-ethyl-3-[3-dimethylaminopropyl] carbodiimide hydrochloride) was co-injected with 20 nM (sulfo-NHS) (*N*-hydroxysulfosuccinimide) (30 μl/min; 5 min) over the surface followed by the injection of 25 μg/ml antibody in 10 mM Na-acetate pH 4.5 buffer (30 μl/min; 5 min). Deactivation of the surface was achieved by injecting 1M ethanolamine pH 8.5 (30 μl/min; 5 min). The running buffer contained 20 mM HEPES, 150 mM NaCl, 5mM CaCl_2_, 0.05% Triton X-100, pH 7.4. For calcium free experiments a similar buffer was used containing 0.1 mM EDTA instead of 5mM CaCl_2_. For the binding assays TFMI-3_HA was injected to yield a 50–80 response unit (RU). Activated MASP-3 was injected onto the chip at 50 nM, 25 nM, 10 nM, 5 nM and 0 nM concentration simultaneously at a flow rate of 25 μl/min. The association phase was set to 420 sec, while dissociation was monitored for 600 sec. The double referenced data were globally fitted to the 1:1 Langmuir binding model.

Binding of zymogen MASP-3cf to TFMI-3 was tested but even at 10 μM injected zymogen did not generate binding signal.

### Human pro-FD and Cy3-labeled pro-FD

Pro-FD, carrying the APPRGR N-terminal propeptide and a His_6_-tag at the C-terminus, was expressed in insect cells and purified to homogeneity as described^27^. Pro-FD was labeled with Cy3-NHS ester (GE Healthcare) and purified as described^27^. Fractions of Cy3-pro-FD containing only one dye molecule per protein molecule were selected in order to obtain sharp bands on SDS-PAGE. Cy3-pro-FD was concentrated to about 1 mg/ml and stored frozen in aliquots.

### Cleavage of Cy3-pro-FD in normal human plasma or serum

Plasma and serum samples from 10 healthy donors were collected by vein puncture after informed consent separately into four different S-Monevette tubes (Sarstedt) prepared with Na_3_-citrate, K_3_-EDTA, recombinant hirudin, or clot activator (silica). Samples of the same type were combined, aliquoted and stored at −80 °C until use. Each aliquot was thawed only once. 50 μL of plasma or serum was supplemented with TFMI-3 at zero, 100 nM, or 1000 nM final concentration added in less than 1 μl causing minimal dilution. After 10 min pre-incubation with TFMI-3 at room temperature 5 μl of Cy3-pro-FD^27^ was added resulting in 91 μg/ml (3.5 μM) final concentration. The mixtures were incubated at 37 °C for 24 hours. Aliquots were withdrawn in every hour in the first 8–10 hours then a final sample was collected after 24 hours. Samples were immediately mixed with reducing SDS-PAGE sample buffer, heated (95 °C, 1 min) and frozen until analysis. Samples were run on 12.5% gels, and the gels were scanned with a Typhoon laser scanner (GE Healthcare). Band intensities were quantified by densitometry.

### Ethics statement

Methods using human blood samples were carried out in accordance with the approved guidelines of the University of Debrecen. Experimental protocols were approved by the local ethics committee of the University of Debrecen. Informed consent was obtained for the isolation of peripheral venous blood from the donors.

## Additional Information

**How to cite this article**: Dobó, J. *et al*. MASP-3 is the exclusive pro-factor D activator in resting blood: the lectin and the alternative complement pathways are fundamentally linked. *Sci. Rep.* **6**, 31877; doi: 10.1038/srep31877 (2016).

## Supplementary Material

Supplementary Information

## Figures and Tables

**Table 1:** Equilibrium inhibitory constants (K_I_) of TFMI-3 and the parent molecule TFPI-D2 on MASPs.

Protease	K_I_(nM)	
	TFMI-3	TFPI-D2
active MASP-1cf	no inhibition	no inhibition
active MASP-2cf	^*^75 000 ± 3 000	^*^318 ± 11
active MASP-3cf	^*^11.1 ± 1.4	no inhibition

^*^Average of 3 repeats (±SD).

**Table 2:** Proteins identified with affinity purification using TFMI-3_HA.

Protein	UniProt ID	Number of unique tryptic peptides found by MS					
		Beads only		TFMI-3_K135E_HA		TFMI-3_HA	
		5′ elution	90′ elution	5′ elution	90′ elution	5′ elution	90′ elution
Ficolin-3	O75636-1	0	0	0	0	2	7
MASP-3	P48740-2	0	0	0	0	0	5

**Table 3:** Half-life and cleavage ratio of labeled pro-FD in the absence or presence of TFMI-3.

Sample	TFMI-3 concentration (nM)	half-life	cleavage ratio at 24 h^d^
citrated plasma^a^	0	4.3 ± 0.3 h^b^	≥95%
	100	≥2 days^c^	~25%
	1000	≥1 week^c^	≤10%
EDTA plasma^a^	0	3.8 ± 0.3 h^b^	≥95%
	1000	≥1 week^c^	≤10%
hirudin plasma^a^	0	2.3 ± 0.3 h^b^	≥95%
	1000	≥1 week^c^	≤10%
serum^a^	0	2.2 ± 0.3 h^b^	≥95%
	1000	~40 h^c^	~35%

^a^Pooled sample from 10 individuals.

^b^Average of 3 repeats (±SD).

^c^Calculated from the cleavage ratio at 24 h assuming exponential decay. These experiments were also carried out multiple times, but these values can be considered only as estimates because of the 24-h end-point of the experiments.

^d^Estimated from the band intensities determined by densitometry.
