# Supplementary material for: MASP-3 is the exclusive pro-factor D activator in resting blood: the lectin and the alternative complement pathways are fundamentally linked
Source: Sci Rep. 2016 Aug 18;6:31877. doi: 10.1038/srep31877 (PMC4989169; doi:10.1038/srep31877)
Supplement: Supplementary Information [file srep31877-s1.pdf]

Supplementary Information for

MASP-3 is the exclusive pro-factor D activator in resting blood: the lectin and the alternative complement pathways are fundamentally linked

József Dobó[1,*], Dávid Szakács[2,*], Gábor Oroszlán[1], Elod Kortvely[3], Bence Kiss[2], Eszter Boros[2], Róbert Szász[4], Péter Závodszky[1], Péter Gál[1], Gábor Pál[2]

[1] Institute of Enzymology, Research Centre for Natural Sciences, Hungarian Academy of Sciences, Magyar tudósok körútja 2, H-1117, Budapest, Hungary

[2] Department of Biochemistry, Eötvös Loránd University, Pázmány Péter sétány 1/C, H-1117, Budapest, Hungary

[3] Institute for Ophthalmic Research, University of Tübingen, Röntgenweg 11, 72076 Tübingen, Germany

[4] Department of Hematology, Institute of Internal Medicine, University of Debrecen, Nagyerdei krt. 98, H-4032, Debrecen, Hungary

[*] These authors contributed equally to this work.

Correspondence may be addressed to J.D. (e-mail: dobo.jozsef@ttk.mta.hu) or G.P. (e-mail: gabor.pal@ttk.elte.hu)

Table S1

Amino acid and DNA sequences of the inhibitory loops of MASP-3-binding TFPI-D2 variants identified by phage display selection. All DNA sequences are unique indicating independent selection events.

	P3	P2	P1	P1'	P2'	P3'	P4'	DNA sequence
1	I	C	K	I	L	L	I	ATTTGCAAGATTCTGCTTATT
2	I	C	K	I	L	L	I	ATTTGCAAGATTTTGCTTATT
3	I	C	K	L	F	F	L	ATTTGCAAGCTTTTTTTTCTT
4	I	C	K	L	F	F	V	ATTTGCAAGCTGTTTTTTGTT
5	I	C	K	L	L	F	L	ATTTGCAAGCTTCTTTTTCTT
6	I	C	R	L	F	F	I	ATTTGCCGGCTTTTTTTTATT
7	I	C	R	L	F	F	I	ATTTGCCGTTTGTTTTTTATT
8	I	C	R	L	F	F	V	ATTTGCAGGCTGTTTTTTGTT
9	I	C	R	L	F	F	V	ATTTGCCGTCTGTTTTTTGTG
10	I	C	R	L	F	F	V	ATTTGCCGTTTGTTTTTTGTT
11	I	C	R	L	L	F	I	ATTTGCAGGCTTCTGTTTATT
12	I	C	R	L	L	F	I	ATTTGCAGGTTGCTTTTTATT
13	M	C	K	L	F	F	I	ATGTGCAAGCTGTTTTTTATT
14	R	C	K	L	F	F	I	CGTTGCAAGCTTTTTTTTATT
15	R	C	K	L	F	F	V	AGGTGCAAGCTTTTTTTTGTT
16	R	C	R	I	L	F	I	CGTTGCCGTATTCTGTTTATT
17	R	C	R	L	F	F	A	AGGTGCAGGTTGTTTTTTGCT
18	R	C	R	L	F	F	I	AGGTGCAGGCTTTTTTTTATT
19	R	C	R	L	F	F	I	AGGTGCCGTTTGTTTTTTATT
20	R	C	R	L	F	F	L	AGGTGCAGGTTGTTTTTTTTG
21	R	C	R	L	F	F	L	CGGTGCCGGCTGTTTTTTTTG
22	R	C	R	L	F	F	V	AGGTGCCGGTTGTTTTTCGTG
23	R	C	R	L	F	F	V	CGGTGCAGGCTTTTTTTTGTG
24	R	C	R	L	F	F	V	CGTTGCCGTCTTTTTTTTGTG
25	R	C	R	L	L	F	I	AGGTGCAGGCTGCTGTTTATT
26	R	C	R	L	L	F	I	CGGTGCAGGTTGCTTTTTATT
27	R	C	R	L	L	F	I	CGTTGCCGTTTGTTGTTTATT
28	V	C	K	I	L	L	I	GTTTGCAAGATTTTGCTTATT
29	V	C	K	L	F	F	I	GTTTGCAAGTTGTTTTTCATT
30	V	C	K	L	F	F	V	GTGTGCAAGCTTTTTTTTGTT
31	V	C	R	L	F	F	I	GTTTGCCGGCTGTTTTTTATT

Table S2

Proteins detected to bind to TFMI-3_HA and their corresponding tryptic peptides

Protein	UniProt ID	peptide sequence	probability
Ficolin-3	O75636-1	(K)GEPGDPVNLLR(C)	100%
		(R)QDGSVDFFR(S)	100%
		(R)AGFGNQESEFWLGNENLHqLTLQGNWELR(V)	100%
		(R)AGFGNQESEFWLGNENLHQLTLQGNWELR(V)	91%
		(R)LLGEVDHYQLALGK(F)	100%
		(K)FSEGTAGDSLSLHSGR(P)	100%
		(R)YAVSEAAAHK(Y)	100%
		(K)YGIDWASGR(G)	100%
MASP-3	P48740-2	(R)TGVITSPDFPNPYPK(S)	99%
		(R)RDTTVIPVSK(E)	88%
		(R)DTTVIPVSK(E)	100%
		(K)EHVTVYLGLHDVR(D)	100%
		(R)TLSDVLQYVK(L)	100%
		(K)QVYGVYTK(V)	99%

Threshold values for identification were >80% and >95% at peptide and protein levels, respectively
Red color indicates tryptic peptides of MASP-3 from a region unique for this splice variant
q indicates deamidation of a Q residue (+1 Da)

Table S3

Sequences of all synthetic genes and oligonucleotides used in the study.

The function of DNA primers and synthetic genes	The sequence of DNA primers and synthetic genes
The TFPI D2 synthetic gene. The flanking Ser/Gly linker coding regions are in italic, while the Kpn2I and SacI restriction sites are underlined.	*TCCGGAGGCTCGGGC*AAACCGGACTTCTGCTTCCTGGAAGAAGAC<br>CCGGGTATCTGCCGTGGTTACATCACCCGTTACTTCTACAACAAC<br>CAGACCAAACAGTGCGAACGTTTCAAATACGGTGGTTGCCTGGGT<br>AACATGAACAACTTCGAAACCCTGGAAGAATGCAAAAACATCTGC<br>GAAGACGGT*GGCGGCAGCGGCGGCAGCGGCGGGAGCTC*
The TFPI D2 P3-P4' STOP primer. The STOP codons are underlined.	CCTGGAAGAAGACCCGGGTTAATGCTAATAATAATAATAACGTTA<br>CTTCTACAACAACCAGACC
The TFPI D2 P3-P4' library primer. The degenerated NNK codons are underlined.	CCTGGAAGAAGACCCGGGTNNKTGCNNKNNKNNKNNKNNKCGTTA<br>CTTCTACAACAACCAGACC
The TFMI-3 mutagenesis primer. The mutation-representing codons are underlined.	CCTGGAAGAAGACCCGGGTATCTGCAAACTGTTCTTCATCCGTTA<br>CTTCTACAACAACCAGACC
The synthetic gene coding for the N-terminally His$_6$-tagged, C3S, C81S, C86S S100A4 followed by the TEV protease cleavage site and a MCS region. The region coding for the His$_6$-tag is in italic and the region coding for the TEV protease cleavage site is in bold, while the sites of restriction endonucleases are underlined.	CCATGGGC*CATCACCATCACCATCAC*GCTAGCATGGCGTCCCCTC<br>TGGAGAAGGCCCTGGATGTGATGGTGTCCACCTTCCACAAGTACT<br>CGGGCAAAGAGGGTGACAAGTTCAAGCTCAACAAGTCAGAACTAA<br>AGGAGCTGCTGACCCGGGAGCTGCCCAGCTTCTTGGGGAAAAGGA<br>CAGATGAAGCTGCTTTCCAGAAGCTGATGAGCAACTTGGACAGCA<br>ACAGGGACAACGAGGTGGACTTCCAAGAGTACTGTGTCTTCCTGT<br>CCTCCATCGCCATGATGTCTAACGAATTCTTTGAAGGCTTCCCAG<br>ATAAGCAGCCCAGGAAGAAAGTCGAC*G***ACTACGACATCCCGACTA**<br>**CCGAAAACCTGTACTTCCAGGGA**TCCCATATGTATGGTACCCGAC<br>TCGAG
The primer providing all TFPI D2 variants with a C-terminal HA-tag. The restriction endonuclease cleavage sites are underlined. The region coding for the HA tag is in italic and the region hybridizing to the TFPI D2 gene is in bold.	TGACCTCGAGTTATTA*CGCATAATCCGGCACATCATATGGGTAAG*<br>*AACCGCCGCTGCC***ACCGTCTTCGCAGATG**
Forward primer to introduce the P1 lysine to glutamate mutation into the TFMI-3 gene using overlap extension PCR. The bases introducing the mutation are underlined.	GGTATCTGCGAACTGTTCTTCATCCG
Reverse primer to introduce the P1 lysine to glutamate mutation into the TFMI-3 gene using overlap extension PCR. The bases introducing the mutation are underlined.	GAAGAACAGTTCGCAGATACCCGGG

Figure S1

The sequence of the MASP-3 precursor and the identified peptides

TFMI-3_HA was incubated with plasma and collected by anti-HA magnetic beads. Only two proteins were found to be exclusive to the functional TFMI-3-HA bait, the targeted protease MASP-3 and ficolin-3. Omission of the inhibitor (beads only) or using its nonfunctional point mutant variant (TFMI-3_K135E_HA) were used as controls. Four out of the five identified tryptic peptides of MASP-3 (green boxes) reside within a region found exclusively in MASP-3 (pink) among the splice variant products of the *MASP-1* gene. The signal peptide is underlined.

```
MRWLLLYYAL CFSLSKASAH TVELNNMFGQ IQSPGYPDSY PSDSEVTWNI
TVPDGFRIKL YFMHFNLESS YLCEYDYVKV ETEDQVLATF CGRETTDTEQ
TPGQEVVLSP GSFMSITFRS DFSNEERFTG FDAHYMAVDV DECKEREDEE
LSCDHYCHNY IGGYYCSCRF GYILHTDNRT CRVECSDNLF TQRTGVITSP
DFPNPYPKSS ECLYTIELEE GFMVNLQFED IFDIEDHPEV PCPYDYIKIK
VGPKVLGPFC GEKAPEPIST QSHSVLILFH SDNSGENRGW RLSYRAAGNE
CPELQPPVHG KIEPSQAKYF FKDQVLVSCD TGYKVLKDNV EMDTFQIECL
KDGTWSNKIP TCKIVDCRAP GELEHGLITF STRNNLTTYK SEIKYSCQEP
YYKMLNNNTG IYTCSAQGVW MNKVLGRSLP TCLPECGQPS RSLPSLVKRI
IGGRNAEPGL FPWQALIVVE DTSRVPNDKW FGSGALLSAS WILTAAHVLR
SQRRDTTVIP VSKEHVTVYL GLHDVRDKSG AVNSSAARVV LHPDFNIQNY
NHDIALVQLQ EPVPLGPHVM PVCLPRLEPE GPAPHMLGLV AGWGISNPNV
TVDEIISSGT RTLSDVLQYV KLPVVPHAEC KTSYESRSGN YSVTENMFCA
GYYEGGKDTC LGDSGGAFVI FDDLSQRWVV QGLVSWGGPE ECGSKQVYGV
YTKVSNYVDW VWEQMGLPQS VVEPQVER
```

Figure S2

Cleavage rate of pro-FD by MASP-3cf in the absence and presence of Ca^{2+}

Pro-FD (18 μM) was incubated in the presence of 30 nM ($[E]_T$) MASP-3cf at 37 °C for up to 2 hours in 140 mM NaCl, 50 mM HEPES, containing either 2 mM $CaCl_2$, or 0.1 mM EDTA. Aliquots were withdrawn at the indicated time points, and the reaction was stopped by dilution and placing the samples to ice. Pro-FD and FD were separated by analytical cation exchange chromatography and the apparent first order rate constants (k_{obs}) were determined as described (8). Representative experiments of 2 parallels are shown. The $k_{obs}/[E]_T$ values represent the average (± range) obtained from the 2 parallel experiments. The obtained values ($3.2 \pm 0.5 \times 10^3$ M^{-1} s^{-1} in the presence of Ca^{2+}, and $3.3 \pm 0.3 \times 10^3$ M^{-1} s^{-1} in the absence of Ca^{2+}) are similar to the value ($4.7 \pm 1.2 \times 10^3$ M^{-1} s^{-1}) determined earlier with different batches of the same proteins under slightly different conditions (8).

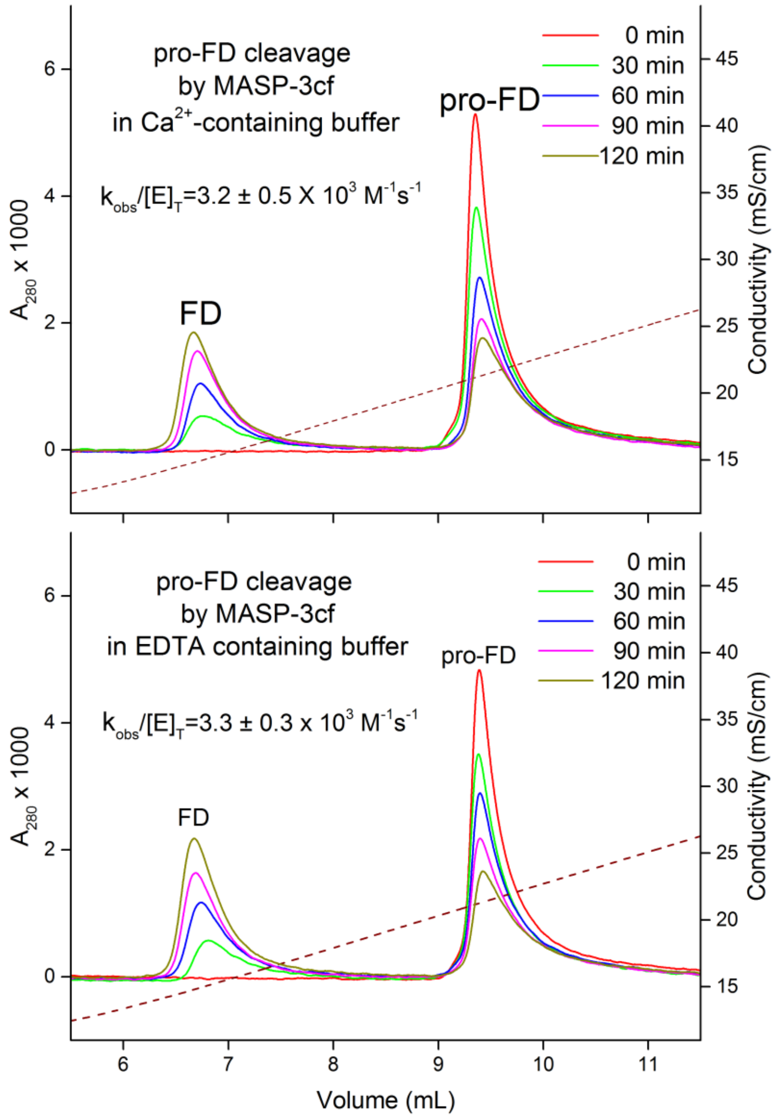

Figure S3

DNA and protein sequence of the TFPI-D2 – p8 fusion gene constructed for phage display

The DNA and protein sequence of the TFPI-D2 – p8 fusion gene. Restriction endonuclease cleavage sites enabling the modular modification of the gene are indicated. The Flag epitope tag, the TFPI D2 and the p8 coat protein are highlighted. The protein starts with an N-terminal periplasmic signal and contains Ser/Gly linkers between the functional modules.

```
     NdeI
  1  CATATGAAAA TAAAAACAGG TGCACGCATC CTCGCATTAT CCGCATTAAC
     H  M  K  I   K  T  G   A  R  I   L  A  L  S   A  L  T

                          NheI
 51  GACGATGATG TTTTCCGCCT CGGCGCTAGC ATCGGATTAT AAAGACGATG
      T  M  M   F  S  A  S   A  L  A   S  D  Y   K  D  D  D  Flag-tag

           HindIII         BspEI
101  ATGACAAAGC TTCCGGCGGG TCCGGAGGCT CGGGCAAACC GGACTTCTGC
       D  K  A   S  G  G   S  G  G  S   G  K  P   D  F  C

151  TTCCTGGAAG AAGACCCGGG TATCTGCCGT GGTTACATCA CCCGTTACTT
     F  L  E  E   D  P  G   I  C  R   G  Y  I  T   R  Y  F

201  CTACAACAAC CAGACCAAAC AGTGCGAACG TTTCAAATAC GGTGGTTGCC
      Y  N  N   Q  T  K  Q   C  E  R   F  K  Y   G  G  C  L

251  TGGGTAACAT GAACAACTTC GAAACCCTGG AAGAATGCAA AAACATCTGC
       G  N  M   N  N  F   E  T  L  E   E  C  K   N  I  C   TFPI D2

                                   SacI
301  GAAGACGGTG GCGGCAGCGG CGGCAGCGGC GGGAGCTCCA GCGCCGAGGG
     E  D  G  G   G  S  G   G  S  G   G  S  S  S   A  E  G

351  TGACGATCCC GCAAAAGCGG CCTTTAACTC CCTGCAAGCC TCAGCGACCG
      D  D  P   A  K  A  A   F  N  S   L  Q  A   S  A  T  E

401  AATATATCGG TTATGCGTGG GCGATGGTTG TTGTCATTGT CGGCGCAACT
       Y  I  G   Y  A  W   A  M  V  V   V  I  V   G  A  T   p8 coat protein

                                                 XbaI
451  ATCGGTATCA AGCTGTTTAA GAAATTCACC TCGAAAGCAA GCTAATAATC
     I  G  I  K   L  F  K   K  F  T   S  K  A  S   *

501  TAGA
```

Figure S4

DNA and protein sequence of the S100A4 – TFMI-3 fusion gene constructed for expression

The DNA and protein sequence of the S100A4 – TFMI-3 fusion construct shows the S100A4, the TEV protease cleavage site and the TFMI-3 modules highlighted. Restriction endonuclease cleavage sites enabling modular modification of the gene are also indicated. The protein starts with an N-terminal His_6-tag for purification on immobilized metal affinity chromatography.

```
      NcoI                          NheI
  1  ACCATGGGCC ATCACCATCA CCATCACGCT AGCATGGCGT CCCCTCTGGA
        M  G  H   H  H  H   H  H  A   S  M  A  S   P  L  E

 51  GAAGGCCCTG GATGTGATGG TGTCCACCTT CCACAAGTAC TCGGGCAAAG
      K  A  L   D  V  M  V   S  T  F   H  K  Y   S  G  K  E

101  AGGGTGACAA GTTCAAGCTC AACAAGTCAG AACTAAAGGA GCTGCTGACC
       G  D  K   F  K  L   N  K  S  E   L  K  E   L  L  T

151  CGGGAGCTGC CCAGCTTCTT GGGGAAAAGG ACAGATGAAG CTGCTTTCCA
     R  E  L  P   S  F  L   G  K  R   T  D  E  A   A  F  Q

201  GAAGCTGATG AGCAACTTGG ACAGCAACAG GGACAACGAG GTGGACTTCC
      K  L  M   S  N  L  D   S  N  R   D  N  E   V  D  F  Q

251  AAGAGTACTG TGTCTTCCTG TCCTCCATCG CCATGATGTC TAACGAATTC
       E  Y  C   V  F  L   S  S  I  A   M  M  S   N  E  F    S100A4

301  TTTGAAGGCT TCCCAGATAA GCAGCCCAGG AAGAAAGTCG ACGACTACGA
     F  E  G  F   P  D  K   Q  P  R   K  K  V  D   D  Y  D    TEV site

                                           BamHI
351  CATCCCGACT ACCGAAAACC TGTACTTCCA GGGATCCAAA CCGGACTTCT
      I  P  T   T  E  N  L   Y  F  Q   G  S  K   P  D  F  C

401  GCTTCCTGGA AGAAGACCCG GGTATCTGCA AACTGTTCTT CATCCGTTAC
       F  L  E   E  D  P   G  I  C  K   L  F  F   I  R  Y

451  TTCTACAACA ACCAGACCAA ACAGTGCGAA CGTTTCAAAT ACGGTGGTTG
     F  Y  N  N   Q  T  K   Q  C  E   R  F  K  Y   G  G  C

501  CCTGGGTAAC ATGAACAACT TCGAAACCCT GGAAGAATGC AAAAACATCT
      L  G  N   M  N  N  F   E  T  L   E  E  C   K  N  I  C    TFMI-3

                    HindIII    XhoI
551  GCGAAGACGG TTAATAAGCT TGGCACTCGA GATCCGGCTG CTAACAAAGC
       E  D  G   *
```
